# Tobacco carcinogen NNK promotes pancreatic cancer proliferation via LINC00857/β-catenin

**DOI:** 10.18332/tid/203455

**Published:** 2025-04-29

**Authors:** Cancan Zhou, Ruiqi Cao, Qiqi Wang, Jiantao Mo, Weikun Qian, Zhengyuan Feng, Shengzhan Zhang, Xin Chen, Jie Hao, Qingyong Ma, Zheng Wu, Zheng Wang

**Affiliations:** 1 Department of Hepatobiliary Surgery, The First Affiliated Hospital of Xi’an Jiaotong University, Xi’an, China; 2 Pancreas Center, Xi’an Jiaotong University, Xi’an, China; 3 Department of Otorhinolaryngology-Head and Neck Surgery, The First Affiliated Hospital of Xi’an Jiaotong University, Xi’an, China

**Keywords:** pancreatic cancer, nicotine-derived nitrosamine ketone, LINC00857, β-catenin

## Abstract

**INTRODUCTION:**

Smoking is a key risk factor for pancreatic cancer (PC). Nicotine-derived nitrosamine ketone (NNK), a major tobacco smoke constituent, has been shown to promote cancer growth, but its specific role in PC progression remains unclear. While long non-coding RNA LINC00857 (lnc RNA) is implicated in cancer progression, its regulation by NNK is unknown. This study aims to investigate whether NNK can drive PC growth and elucidate the underlying mechanisms.

**METHODS:**

Employing an experimental methodology, this investigation treated human pancreatic cancer cell lines (CFPAC-1 and Panc-1) with NNK and utilized various assays (CCK-8, colony formation, and EdU cell proliferation) to assess the effects on cell proliferation. The interplay between LINC00857 expression profiles, PC, and smoking was systematically investigated through cross-database bioinformatic interrogation encompassing public resources and institutional biobank data. Experiments were performed to knock down LINC00857 in PC cells using siRNA technology. We used Western blotting and quantitative real-time PCR (qRT-PCR) to assess β-catenin expression and elucidate the mechanism by which the tobacco carcinogen NNK promotes PC formation.

**RESULTS:**

Some evidence that NNK enhanced the proliferative capacity of PC cells was found. Bioinformatic analysis of public databases, combined with data from our center's database, revealed that LINC00857 was up-regulated in PC and correlated with smoking. Moreover, we discovered that knockdown of LINC00857 inhibited PC cell proliferation, with β-catenin identified as a potential downstream molecule. Importantly, after LINC00857 knockdown, we observed suppression of NNK-induced β-catenin upregulation at both protein and transcriptional levels.

**CONCLUSIONS:**

NNK potentially induces PC progression through the LINC00857/β-catenin axis. These findings provide new perspectives on the mechanisms of PC progression and highlight the clinical relevance of smoking cessation for preventing PC.

## INTRODUCTION

Despite significant advancements in our understanding, PC remains a devastating disease with a poor prognosis, the outcome remains grim. Research has identified several risk factors associated with the progression of PC, including chronic pancreatitis, diabetes mellitus, and genetic predispositions alongside lifestyle choices^1^. Unlike other risk factors, lifestyle factors are modifiable, which can contribute to the prevention of PC. Among lifestyle risk factors of PC are primarily cigarette smoking and alcohol consumption. Current studies indicate that smoking increases the risk of PC^2^, accelerates PC development^3^, shortens the survival time of PC patients^4^, and increases the total mortality of PC patients^5^. Although numerous studies have been conducted, the relationship between cigarette smoking and the progression of PC is yet to be revealed^4^.

Cigarette smoke, a well-established risk factor for PC, is composed of a complex mixture of substances and demonstrates a sophisticated carcinogenic mechanism^6,7^. Nicotine, a major component of cigarette smoke, is closely associated with the malignant progression of various tumors^8,9^, after being converted into nitrosamines. Among the nicotine-derived nitrosamine ketone, the most important carcinogen is NNK, possessing high toxicity and carcinogenicity^10^. Existing research has demonstrated that NNK can influence the activity of different components in the tumor microenvironment, such as HIF-1α and β2-AR, thereby promoting the growth of tumor cells. Additionally, studies have indicated a negative correlation between NNK and the total survival rate of PC patients^11,12^. However, the impact of NNK on PC cells and its mechanism remains unclear.

Previous studies have identified lncRNAs longer than 200 nucleotides as key regulators of various cancers, including PC^13^. Simultaneously, research has also indicated that lncRNAs are associated with exposure to chemical carcinogens, including NNK^14^. LINC00857 is a type of lncRNA that has been shown to enhance the epithelial-mesenchymal transition (EMT) of hepatocellular carcinoma cells^15^ and mediate tumor progression in lung cancer patients^16^. Additionally, our preceding studies have suggested that LINC00857 is connected to the progression of PC^17,18^; however, the relationship between NNK and LINC00857 has never been studied.

The Wnt signaling pathway is a critical pathway involved in cell proliferation, differentiation, and migration. Over the past few years, an ever-increasing number of studies have indicated that key molecules in the Wnt signaling pathway, such as β-catenin and MAPK, are tied to different types of cancer, including PC^19^. As research has advanced, it has been discovered that lncRNAs can regulate key factors in this pathway, such as Wnt ligands, Frizzled receptors, and β-catenin^20^. Despite this, it remains uncertain whether LINC00857 can regulate the Wnt pathway.

Within this study, it primarily elucidated the impact of NNK in PC growth and identified LINC00857 as a potentially functional target for its underlying mechanism. This study aims to treat PC cell lines (CFPAC-1 and Panc-1) with NNK and utilize various experimental assays, including CCK-8, colony formation, and EdU cell proliferation assays, to evaluate its effects on cellular proliferation and investigate potential mechanisms.

## METHODS

### Cell culture and treatment

This study follows an experimental research design. CFPAC-1 and Panc-1 human PDAC cell lines were sourced from the certified repository CBTCCCAS at the Chinese Academy of Sciences (Shanghai, China), a WHO-registered cell culture collection. These cell populations were routinely maintained in high-glucose Dulbecco’s Modified Eagle’s Medium (DMEM; Gibco, Thermo Fisher Scientific, USA), supplemented with 10% (v/v) heat-inactivated fetal bovine serum (FBS; Gibco) and 1% penicillin-streptomycin antibiotic mixture. Cell cultures were sustained in a humidified incubator at 37°C with a 5% CO_2_/95% air atmosphere, and the medium was replenished every 48 to 72 hours to maintain the logarithmic growth phase. The tobacco-specific nitrosamine, NNK, with a purity exceeding 98%, was obtained from Toronto Research Chemicals (TRC) in Canada.

### Cell proliferation and colony formation assays

In 96-well microplates containing CFPAC-1/Panc-1 cell monolayers (initial density: 1000 cells/well), continuous exposure to 100 μM NNK was performed over four time intervals (24, 48, 72, and 96 h) in a humidified incubator maintained at 37°C with 5% CO_2_ atmosphere. Post-treatment wells received 10 μL CCK-8 solution followed by 3-h incubation, with subsequent optical density (OD) determination at 450 nm using a multimode detection system. To assess the colony formation capacity, Panc-1 and CFPAC-1 cell lines were plated in culture dishes of diameter 6 cm, at an initial seeding density of 1000 cells per dish. They were then treated with 100 μM NNK for 48 h, followed by a 2-week incubation period. Post-fixation was performed using 4% PFA solution (pH 7.4) for 15 min under ambient conditions (25°C), followed by nuclear counterstaining with 0.1% w/v crystal violet aqueous solution (CAS 548-62-9) for 20 min. This was followed by three washes with distilled water to facilitate the macroscopic quantification of colony formation.

### EdU cell proliferation assay

PC cells were plated in 96-well plates at a density of 10000 cells per well and subsequently exposed to 100 μM NNK for 24 h. Cellular proliferation was quantitatively analyzed using a EdU assay kit (Beyotime, C0071S) as per the manufacturer’s protocol. Fluorescence imaging was conducted using a fluorescence microscope (Nikon A1R/A1 system).

### Bioinformatics analysis

In order to investigate the expression level of LINC00857 in PC, relevant data were downloaded from the TCGA database^21^, including 177 cases of PC and 4 cases of normal pancreatic tissue. First, the expression level of LINC00857 was compared between healthy pancreatic tissue and PC tissue. Subsequently, the PC population was further divided into smoking and non-smoking groups based on smoking status, and the presentation level of LINC00857 was analyzed in each group. Finally, the population with PC with smoking history was categorized into two duration-based cohorts: <30 years and ≥30 years of tobacco exposure. The expression level of LINC00857 was then analyzed in each group. This comprehensive analysis will provide valuable insights into the role of LINC00857 in PC and its association with smoking.

### RNA isolation and qRT–PCR

Total RNA was extracted from CFPAC-1 and Panc-1 cells using the Fastgen2000 RNA Isolation System (Fastgen, Shanghai, China) based on the manufacturer’s protocol. cDNA was synthesized from the isolated RNA using the PrimeScript^™^ RT Reagent Kit (TaKaRa, Dalian, China) under standard thermal cycling conditions. The qRT–PCR analysis was conducted in triplicate using SYBR Green Master Mix (Bio-Rad) on a Bio-Rad CFX96^™^ Real-Time System, with β-actin serving as an endogenous control. Relative measurement of target gene expression was calculated using the comparative threshold cycle (2^-ΔΔCt^) method^18^.

### Western blotting assay

Western blot was performed as described previously^22^. Human pancreatic adenocarcinoma cell lines (CFPAC-1 and Panc-1) were seeded in 6-well plates and cultured for 24 h prior to treatment with 100 μM NNK for 48 h. For gene silencing experiments, cells were transiently transfected with siRNA targeting LINC00857, followed by lysis in RIPA buffer supplemented with protease inhibitors. Total protein extracts were quantified via BCA assay, separated by 10% SDS-PAGE, and electrotransferred onto PVDF membranes (Roche, Penzberg, Germany). Membranes were blocked with QuickBlock^™^ Blocking Buffer for 20 min at room temperature, then incubated overnight at 4°C with primary antibodies diluted in TBST. After three washes, membranes were probed with horseradish peroxidase (HRP)-conjugated secondary antibodies (1:5000) for 2 h at 25°C. Protein bands were visualized using an enhanced chemiluminescence (ECL) kit (NCM Biotech, Suzhou, China) on a ChemiDoc XRS System (Bio-Rad, Hercules, CA, USA). Primary antibodies included: anti-β-catenin (1:1000, #9582, Cell Signaling Technology), anti-PCNA (1:2000; 60097-1-Ig, Proteintech, Wuhan, China), anti-CyclinD1 (1:1000; 60186-1-Ig, Proteintech), and anti-β-actin (1:5000; 66009-1-Ig, Proteintech), standing as a loading control.

### Patients and specimens

The tissue samples and surgical operations were gathered by the Department of Hepatobiliary Surgery at the First Affiliated Hospital of the Medical College, Xi’an Jiaotong University. This included PC and their matched normal pancreatic tissues (n=45). The samples were maintained in liquid nitrogen immediately after collection, and total DNA was extracted at a later time. Ethical approval for the trial was obtained from the Internal Research Committee of the First Affiliated Hospital of the Medical College, Xi’an Jiaotong University, China. Written informed consent was acquired from the patients participating in the study.

### Immunofluorescence staining

CFPAC-1 and Panc-1 PC cell lines were plated in 24-well culture plates and permitted to attach for 6 h at 37°C. Subsequently, the cells were fixed with 4% PFA for 15 min. Cellular membranes were permeabilized using permeabilization solution (1% Triton X-100 in PBS) to enhance antibody accessibility. Non-specific binding sites were blocked with 5% bovine serum albumin (BSA; Sigma-Aldrich, Germany) in PBS for 1 h at room temperature. Primary antibodies, diluted in blocking buffer, were used for the samples and incubated at 4°C overnight. Species-matched secondary antibodies conjugated with fluorophores were then introduced and incubated for 1 h at room temperature, protected from light. Nuclear counterstaining was conducted using DAPI (1:10000 dilution) for 15 min in the dark. Finally, fluorescence imaging was conducted using a Nikon A1R/A1 fluorescence microscope.

### Statistical analysis

All statistical analyses were carried out with GraphPad Prism 9.5 (GraphPad Software, USA). Data are expressed as mean ± standard deviation (SD) from three independent experiments. Comparisons of two groups were analyzed by two-tailed Student’s t test, while multi-group comparisons were evaluated using one-way ANOVA. Bioinformatics analyses were conducted in R (version 4.4.2), with Pearson correlation analysis applied for gene correlation assessments. Statistical significance was defined as p<0.05.

## RESULTS

### NNK promotes the proliferation of PC cells

**Figure 1:**
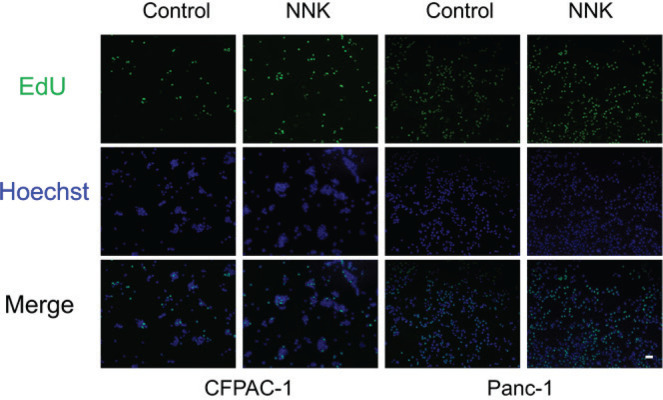
Proliferation of pancreatic cancer cells induced by NNK was detected by EdU assay

Cigarette smoking is one of the risk factors of PC while NNK is the main cancerogenic substance of smoking. To elucidate the effect of NNK on PC, we first performed CCK-8 assay. The concentration of NNK (100 μM) used in our study was selected based on our previous study^23^. As shown in Supplementary file Figures 1A and 1B, when treating with 100 μM NNK, PC cell lines CFPAC-1 and Panc-1 exhibited a better growth trend in a time-dependent manner. EdU staining revealed that the NNK group exhibited significantly higher replicative DNA synthesis compared to the control group (Figure 1), indicating enhanced cellular proliferation capacity. Similarly, more colonies were counted in NNK group compared to control group (Supplementary file Figure 1C). Moreover, we detected the expression of PCNA and CyclinD, the markers of proliferation, through western blotting. As shown in Supplementary file Figure 1D, treatment of NNK increased the expression of PCNA and CyclinD. Collectively, these results reveal that the exposure of NNK accelerates the proliferation of PC.

### LINC00857 is overexpressed in PC and is correlated with cigarette smoking

Based on our previous study, LINC00857 plays a cancer-promoting role in PC^18^. Thus, we hypothesized that LINC00857 may be correlated with cigarette smoke and may be regulated by NNK. To validate our hypothesis, we analyzed the data downloaded from TCGA database, and noted that the level of expression of LINC00857 was much higher in PC (n=141) than that in normal pancreas (n=4) (Supplementary file Figure 2A). PC patients were stratified into smoker (n=57) and non-smoker (n=120) groups based on their smoking history. Supplementary file Figure 2B demonstrated that LINC00857 expression levels were elevated in the smoker group relative to the non-smoker group; however, no statistically significant difference was observed between the groups. We also divided the patients with smoking history into two groups according to duration of smoking. As shown in Supplementary file Figure 2C, we could see that the expression of LINC00857 was elevated as the time of smoking time. However, there is no significant difference.

**Figure 2:**
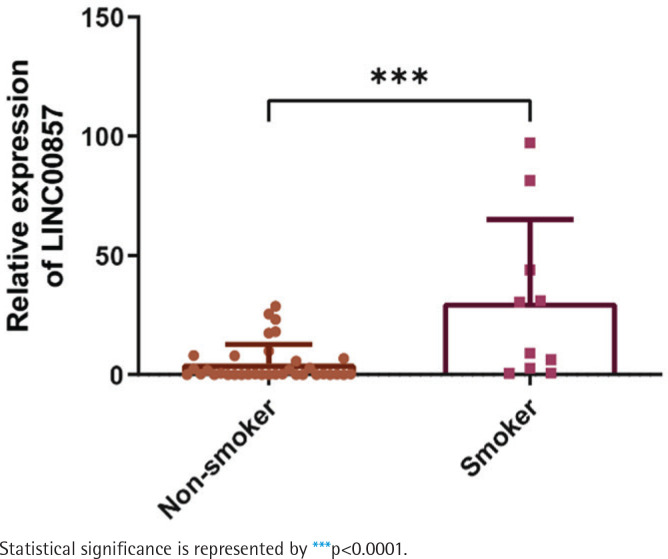
LINC00857 upregulation in smokers with pancreatic cancer

Based on the findings mentioned above, we further collected samples from patients at our center, including those with PC and their paired normal pancreatic tissue (n=45). As the RNA was extracted and qRT-PCR was conducted, we discovered that LINC00857 was significantly expressed in PC tissues as compared to paired normal pancreas (Supplementary file Figure 2D). Again, patients were divided into two groups according to smoking history, and the results showed that smoking increased the expression of LINC00857 and the difference between the smoker group (n=10) and non-smoker group (n=35) was statistically significant (Figure 2). At the same time, we performed fluorescence *in situ* hybridization (FISH) to visualize the location of LINC00857 in human tissue samples. As shown in Supplementary file Figure 2E, we could tell that LINC00857 was much higher in PC tissues than in normal pancreas. As for location, we found that LINC00857 could be detected in both cytoplasm and nucleus in normal pancreas and PC, which was same as we found in PC cells previously^18^. Next, we investigated the expression of LINC00857 in PC cell lines as well as in normal pancreatic ductal epithelial cells, confirming its overexpression in PC (Supplementary file Figure 2F). In line with the expression level in cell lines, CFPAC-1 and Panc-1 cell lines were picked for further studies. Moreover, we treated PC lines with NNK and detected the expression level of LINC00857. The results showed that NNK upregulated the LINC00857 expression (Supplementary file Figure 2G). Taken together, these findings indicate that LINC00857 is overexpressed in PC and its expression level can be upregulated by cigarette smoking.

### Knockdown of LINC00857 suppresses the proliferation of PC cells

**Figure 3:**
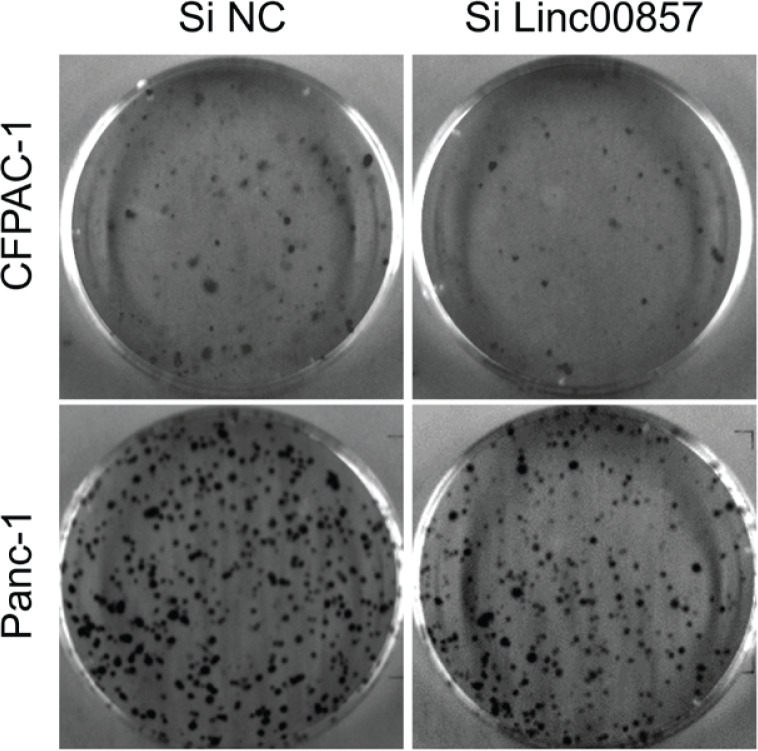
LINC00857-mediated regulation of clonogenic potential in pancreatic cancer cells evaluated by colony formation assay

To explore the role of LINC00857 in PC, we performed more molecular studies. Firstly, we designed SiRNA to knock down the expression of LINC00857 and the effect was verified by qRT-PCR. From Supplementary file Figure 3A, we could see that after transfection of Si LINC00857, the expression level of LINC00857 decreased dramatically compared to Si NC group. After the confirmation of knockdown of LINC00857 by siRNA transfection, we conducted further studies. By CCK-8 assay, we found that knockdown of LINC00857 decreased the growth of PC cells (Supplementary file Figures 3B and 3C). Next, we performed EdU staining and colony formation assay to evaluate the effect of LINC00857 in PC growth. As shown in Figure 3 and Supplementary file Figure 3D, transfection of Si LINC00857 decreased the number of replicative DNA and colony compared to Si NC group. Moreover, we detected the expression level of proliferation markers, PCNA and CyclinD, by Western blotting. The results showed that knockdown of LINC00857 suppressed the expression of PCNA and CyclinD (Supplementary file Figure 3E). The above results indicate that knockdown of LINC00857 suppressed the proliferation of PC cells.

### β-catenin is the potential downstream of LINC00857

**Figure 4:**
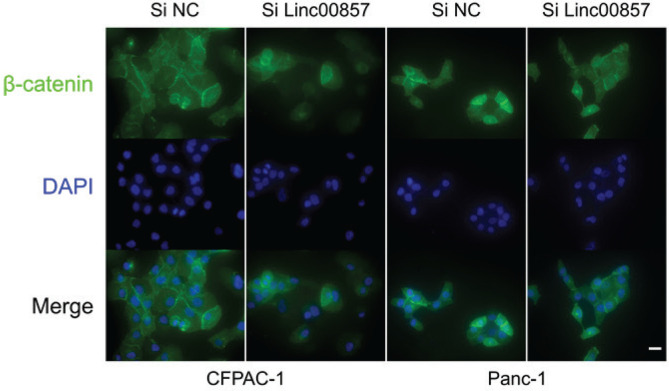
LINC00857-mediated regulation of β-catenin expression in pancreatic cancer cells assessed by immunofluorescence staining

To further investigate the potential downstream effects of LINC00857, we utilized data from TCGA again. As illustrated in Supplementary file Figures 4A and 4B, a comprehensive analysis of TCGA pancreatic adenocarcinoma (PAAD) sequencing data revealed significant positive correlations (FDR-adjusted p<0.05) between LINC00857 expression and key effector molecules of the canonical Wnt/β-catenin signaling pathway. These molecules include β-catenin (CTNNB1, r=0.69), suggesting a potential regulatory role for LINC00857 in Wnt-driven PC progression. Consequently, we examined the impact of LINC00857 knockdown on β-catenin expression. As anticipated, we found that the knockdown of LINC00857 not only decreased β-catenin expression at the mRNA level but also at the protein level (Supplementary file Figures 4C and 4D). The results of IF staining further corroborated these findings (Figure 4). Collectively, these results suggest that LINC00857 functions by targeting β-catenin in PC cells.

### NNK promotes tumor growth by LINC00857/βcatenin signaling pathway in PC

**Figure 5:**
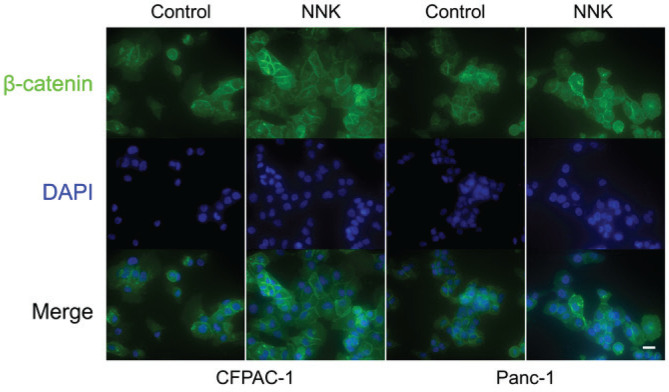
NNK-mediated regulation of β-catenin expression in pancreatic cancer cells assessed by immunofluorescence staining

Although β-catenin is potential downstream of LINC00857; however, whether NNK promotes PC growth by targeting β-catenin remains unknown. We subsequently evaluated β-catenin expression after NNK treatment. Unsurprisingly, we found that exposure to NNK upregulated β-catenin expression at both the mRNA and protein levels (Supplementary file Figures 5A and 5B). Again, we performed IF staining and found that the expression of β-catenin increased when treated with NNK (Figure 5). In summary, these results demonstrated that exposure to NNK could upregulate the expression of β-catenin. To further elucidate whether the upregulation of β-catenin induced by NNK in PC is mediated by LINC00857, additional studies were carried out. We detected the expression of β-catenin by qRT-PCR first. As shown in Supplementary file Figure 5C, we found that the upregulation of β-catenin induced by NNK exposure can be reversed by transfection of Si LINC00857. Similarly, the upregulation of β-catenin induced by NNK could also reversed by Si LINC00857 transfection at protein level (Supplementary file Figure 5D). Moreover, by detecting the expression level of proliferation markers, PCNA and CyclinD, we found that the growth promotion effect of NNK was also inhibited by Si LINC00857 transfection (Supplementary file Figure 5D). To sum up, the above results reveal that NNK-induced upregulation of β-catenin is mediated by LINC00857 and NNK promotes PC growth by LINC00857/β-catenin pathway.

## DISCUSSION

PC is a highly malignant disease associated with known risk factors^24,25^. Smoking has been identified as a risk factor for various tumors, particularly NNK in cigarette smoke, which exhibits high toxicity and carcinogenicity^10,26^. However, the mechanism by which smoking promotes PC proliferation remains unclear. In this study, we discovered that NNK, a component of cigarette smoke, can promote PC tumor growth by upregulating the expression of lncRNA in PC cells, thereby modulating the β-catenin signaling pathway.

Initial studies have identified NNK as a risk factor for lung cancer^27^. Subsequent studies have further revealed significant associations between NNK exposure and an increasing number of cancer types^22,28^. The relationship and mechanisms of action between NNK and PC have also attracted attention. Our prior study established an association between tobacco smoking and PC, demonstrating that cigarette smoke exposure significantly increases the risk of pancreatic carcinogenesis^22,29^. Evidence has shown that NNK promotes the progression of PC by inducing the activation of CREB and subsequently upregulating the expression of GM-CSF^30^. Simultaneously, research has also shed light on the role and mechanisms of NNK in the transformation process from chronic pancreatitis to PC^23^. However, the mechanisms through which NNK promotes the growth of PC are complex and require further exploration. The present study, to our knowledge, is the first to report that NNK can upregulate the expression of the LINC00857 in PC. Our findings demonstrate that NNK can promote the proliferation and clonogenic capacity of PC cells, and we have elucidated the potential underlying regulatory mechanism.

LINC00857, a long non-coding RNA, has been implicated in promoting cell proliferation across multiple cancer types and has been found to be aberrantly overexpressed in PC. According to previous studies, LINC00857 can promote the growth and metastasis of PC cells by regulating the expression of molecules such as MET and FOXM1^18,31^. In the present study, we utilized bioinformatics approaches to analyze the expression of LINC00857 and observed a significant upregulation in PC tissues compared to normal pancreatic tissues. Subsequent analysis indicated that smoking was correlated with elevated expression levels of LINC00857. However, data obtained from public databases did not show statistically significant differences. To further investigate this relationship, we utilized data from our research center. Our findings revealed that the expression levels of LINC00857 were significantly upregulated in PC tissues compared to normal pancreas tissues. Furthermore, we observed that PC patients with a history of smoking exhibited significantly higher expression of LINC00857 compared to their non-smoking counterparts. Then, we applied FISH and confirmed that LINC00857 showed significantly higher in PC tissues than in normal pancreatic tissues. Moreover, LINC00857 was detectable in both the cytoplasmic and nuclear compartments. To further investigate the role of LINC00857, we conducted *in vitro* experiments using PC cell lines and normal pancreatic ductal epithelial cell lines. Our results demonstrated that the expression of LINC00857 was significantly higher in PC cell lines contrast to normal cell lines. Furthermore, its expression was further increased following NNK treatment. To elucidate the functional significance of LINC00857, we employed knockdown experiments and found that suppressing LINC00857 significantly inhibited the proliferation and clonogenic potential of PC cells.

β-catenin, a key regulator of the Wnt signaling pathway, plays a crucial role in cell proliferation^32,33^. Previous studies have shown that dysregulation of the Wnt/β-catenin signaling pathway is closely related to various diseases, and β-catenin mutations are the most common alterations in cancer^1,34^. In this study, through a re-analysis of TCGA data, we discovered a positive correlation between Wnt/β-catenin pathway genes and LINC00857 in PC. Further experiments revealed that knocking down LINC00857 could reduce the mRNA and protein levels of β-catenin. NNK stimulation upregulated the expression of β-catenin, while transfection with siLINC00857 reversed the NNK-induced upregulation of β-catenin. This suggests that LINC00857 may be involved in the occurrence and development of PC by regulating the Wnt/β-catenin signaling pathway. As a controllable social factor, reducing smoking may effectively lower the risk of PC. Additionally, avoiding exposure to harmful substances such as NNK can also decrease the risk of PC. This study provides new insights into the mechanisms of PC occurrence and progression, offering a theoretical basis for the clinical prevention of PC.

### Limitations

This study has limitations that need to be acknowledged. While we identified the regulatory role of NNK in the LINC00857/β-catenin axis, its broader functional impacts beyond cell proliferation remain unresolved. Additionally, our reliance on *in vitro* models (CFPAC-1/Panc-1) limits physiological relevance due to the absence of stromal interactions and the complexity of the tumor microenvironment. Furthermore, bioinformatics analyses based on limited public datasets may introduce bias. Finally, the generalizability of our findings is constrained by the specific experimental conditions; future studies employing diverse models (such as, patient-derived organoids and genetically engineered mice) and expanded clinical cohorts are essential to validate these mechanisms in broader contexts.

## CONCLUSIONS

NNK can promote PC growth, and the regulation of β-catenin by LINC00857 is a potential underlying mechanism. These results provide novel mechanistic insights into the promotion effect of cigarette smoking in PC and highlight the potential preventative implications of tobacco abstinence as a preventative strategy for PC.

## Supplementary Material



## Data Availability

The data supporting this research are available from the authors on reasonable request.
