# Supplementary material for: Tobacco carcinogen NNK promotes pancreatic cancer proliferation via LINC00857/β-catenin
Source: Tob Induc Dis. 2025 Apr 29;23:10.18332/tid/203455. doi: 10.18332/tid/203455 (PMC12039305; doi:10.18332/tid/203455)
Supplement: Supplementary file 1 [file TID-23-50-s1.pdf]

Supplementary file Figure 1. NNK promotes the proliferation of pancreatic cancer cell **A-B** CFPAC-1 and Panc-1 cells were treated with NNK (0, 100 μM) for 24, 48, 72h and 96 h, and cell viability was determined using the CCK-8 assay. **C** The clonogenesis of pancreatic cancer cells in NNK (0, 100 μM) intervention groups was measured using the colony formation assay. **D** Western blotting analysis detected the level of the PCNA and CyclinD1 protein in different treatment groups (0 and 100 μM NNK). Statistical significance is represented by **P<0.001.

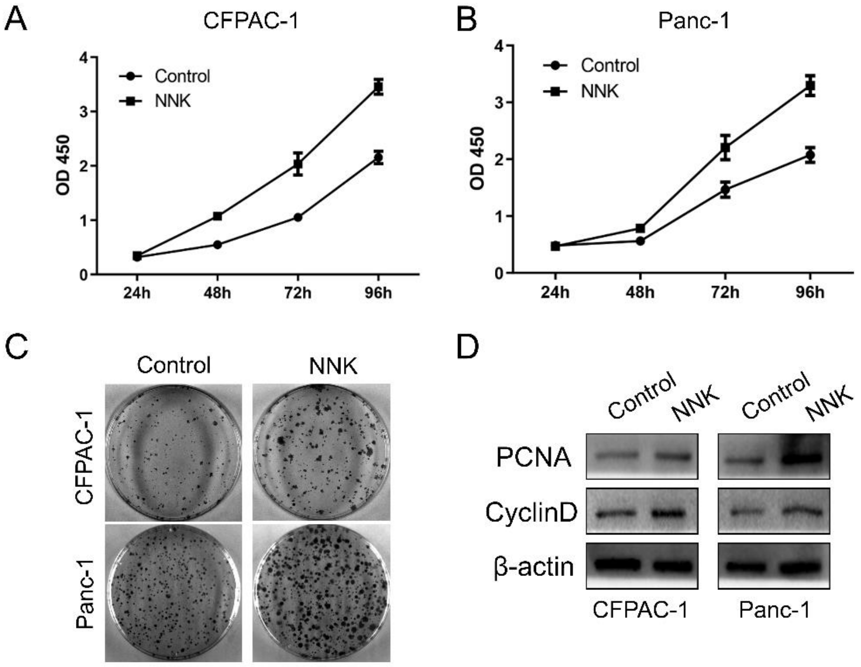

A
CFPAC-1
Control
NNK
OD 450
0
1
2
3
4
24h
48h
72h
96h
B
Panc-1
Control
NNK
OD 450
0
1
2
3
4
24h
48h
72h
96h
C
Control
NNK
CFPAC-1
Panc-1
D
Control
NNK
Control
NNK
PCNA
CyclinD
β-actin
CFPAC-1
Panc-1

Supplementary file Figure 2. LINC00857 is overexpressed in pancreatic cancer and is correlated with cigarette smoking **A** Expression levels of LINC00857 in pancreatic cancer (n=141) and normal tissues (n=4) were analyzed using the TCGA database. **B** The TCGA database was utilized to investigate the expression of LINC00857 in pancreatic cancer patients with and without a history of smoking. **C** The TCGA database was employed to compare the expression levels of LINC00857 between pancreatic cancer patients with a smoking history of over 30 years and those with a smoking history of less than 30 years. **D** Expression levels of LINC00857 in pancreatic cancer (n=10) and normal tissues (n=35) were detected using our center's database samples. **E** The localization of LINC00857 in normal pancreatic tissues and pancreatic cancer tissues was observed using FISH. **G** The mRNA expression levels of LINC00857 in pancreatic cancer cell lines and normal pancreatic ductal epithelial cell lines were detected using qRT-PCR. **F** The mRNA expression levels of LINC00857 were detected using qRT-PCR in different treatment groups (0 and 100 μM NNK). Statistical significance is represented by *P<0.05, **P<0.01 and ***P<0.001.

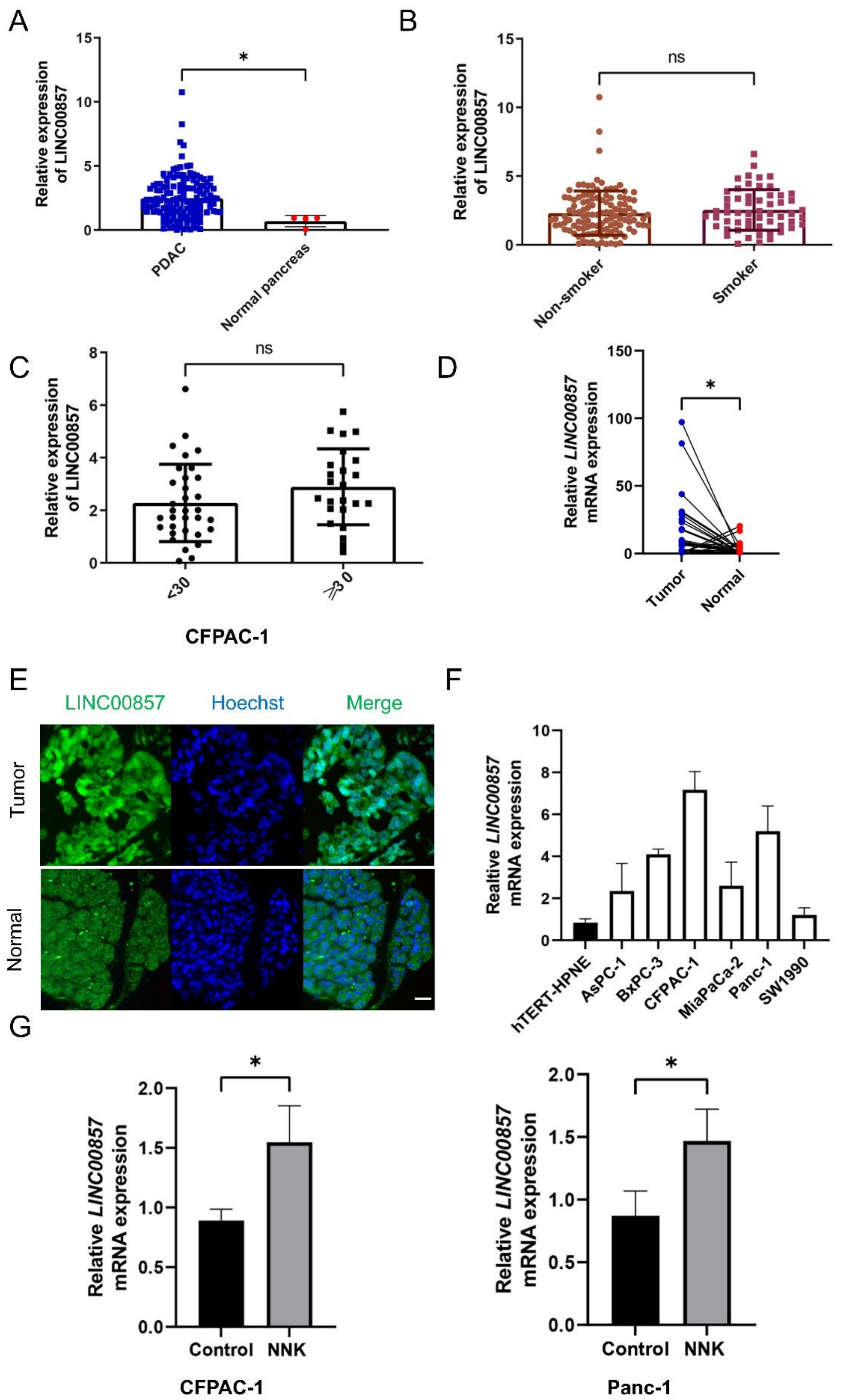

A
B
C
D
E
F
G
Relative expression of LINC00857
PDAC
Normal pancreas
ns
Non-smoker
Smoker
<30
≥30
CFPAC-1
Relative LINC00857 mRNA expression
Tumor
Normal
LINC00857
Hoechst
Merge
Realtive LINC00857 mRNA expression
hTERT-HPNE
AsPC-1
BxPC-3
CFPAC-1
MiaPaCa-2
Panc-1
SW1990
Control
NNK
CFPAC-1
Panc-1

Supplementary file Figure 3. Knockdown of LINC00857 suppresses the proliferation of pancreatic cancer cell **A** CFPAC-1 and Panc-1 cells were transfected with different siRNA interventions (Si NC, Si LINC00857) and the expression levels of LINC00857 were detected using qRT-PCR in both pancreatic cancer cell lines. **B-C** CFPAC-1 and Panc-1 cells were treated with different siRNA interventions (Si NC and Si LINC00857) for 24, 48, 72, and 96 hours, and cell viability was evaluated using the CCK-8 assay. **D** Cell proliferation was assessed using the EdU cell proliferation assay kit. **E** Western blotting analysis was performed to detect the levels of PCNA and CyclinD in different siRNA intervention groups (NC and Si LINC00857). Statistical significance is represented by *$P<0.05$, **$P<0.01$ and ***$P<0.001$.

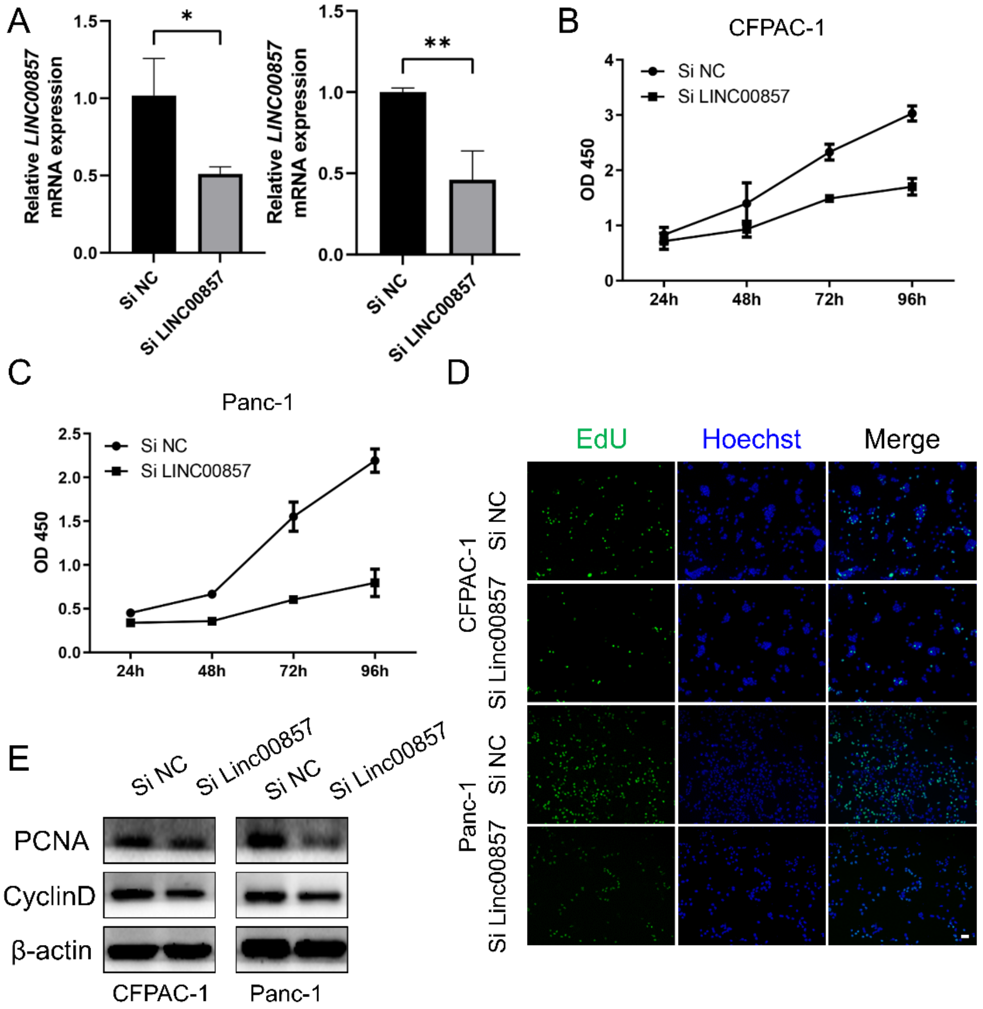
A
Relative LINC00857 mRNA expression
1.5
1.0
0.5
0.0
*
**
Si NC
Si LINC00857
B
CFPAC-1
Si NC
Si LINC00857
OD 450
24h
48h
72h
96h
C
Panc-1
D
EdU
Hoechst
Merge
CFPAC-1
Si Linc00857
Panc-1
E
Si Linc00857
PCNA
CyclinD
β-actin
CFPAC-1
Panc-1

Supplementary file Figure 4. β-catenin is the potential downstream of LINC00857 **A-B** Downstream genes associated with LINC00857 were identified using the TCGA database. **C** CFPAC-1 and Panc-1 cells were transfected with different siRNA interventions (Si NC, Si LINC00857) and the mRNA expression levels of β-catenin were detected using qRT-PCR in both pancreatic cancer cell lines. **D** Western blotting analysis was performed to detect the protein expression levels of β-catenin in CFPAC-1 and Panc-1 cells treated with different siRNA interventions (Si NC, Si LINC00857). Statistical significance is represented by *P<0.05 and **P<0.01.

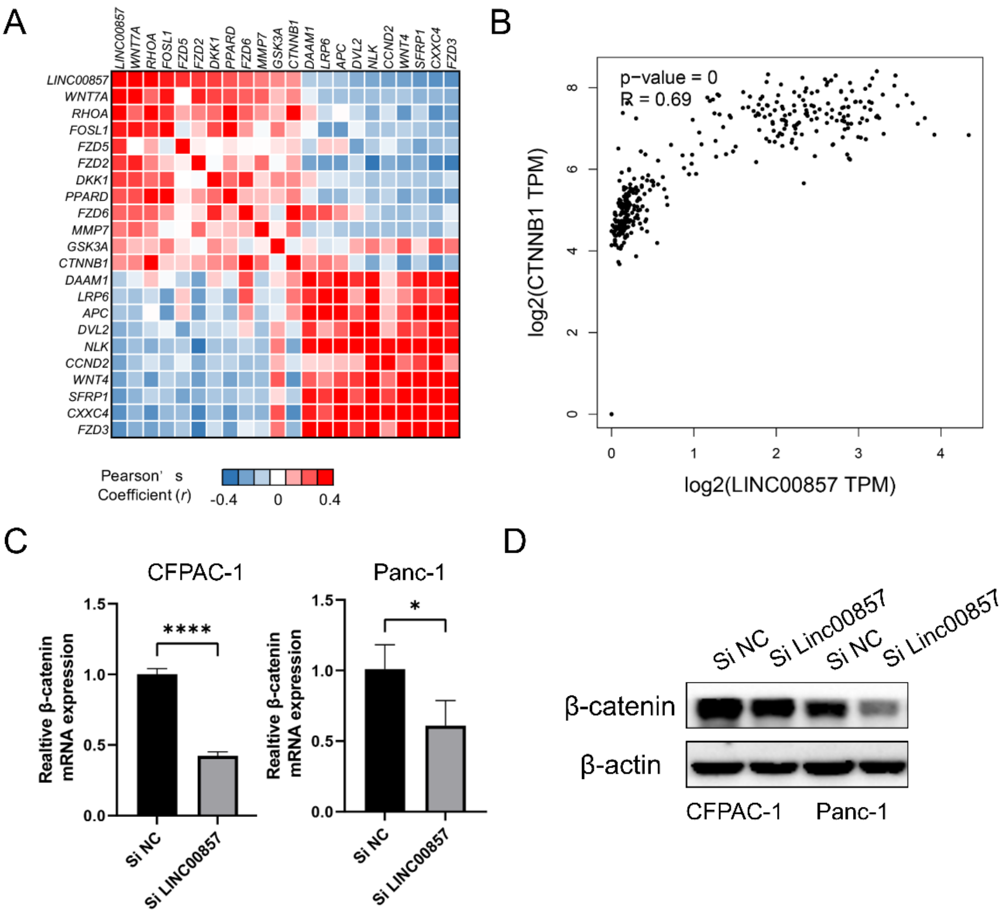
A
B
p−value = 0
R = 0.69
log2(CTNNB1 TPM)
log2(LINC00857 TPM)
Pearson's
Coefficient (r)
-0.4
0
0.4
C
CFPAC-1
Panc-1
Realtive β-catenin mRNA expression
****
*
Si NC
Si LINC00857
D
Si NC
Si Linc00857
β-catenin
β-actin
CFPAC-1
Panc-1

Supplementary file Figure 5. NNK promotes tumor growth by LINC00857/β-catenin signaling pathway in pancreatic cancer **A** CFPAC-1 and Panc-1 cells were treated with NNK (0 and 100 μM) and the mRNA expression levels of β-catenin were detected using qRT-PCR in both pancreatic cancer cell lines. **B** Western blotting analysis was performed to detect the protein expression levels of β-catenin in CFPAC-1 and Panc-1 cells treated with NNK (0 and 100 μM). **C** CFPAC-1 and Panc-1 cells were treated with NNK and siRNA interventions (Si NC, NNK+Si NC, NNK+Si LINC00857) and the mRNA expression levels of β-catenin were detected using qRT-PCR in both pancreatic cancer cell lines. **D** Western blotting analysis was performed to detect the protein expression levels of β-catenin, PCNA, and CyclinD in different treatment groups (Si NC, NNK+Si NC, NNK+Si LINC00857). Statistical significance is represented by *P<0.05 and ****P<0.0001.

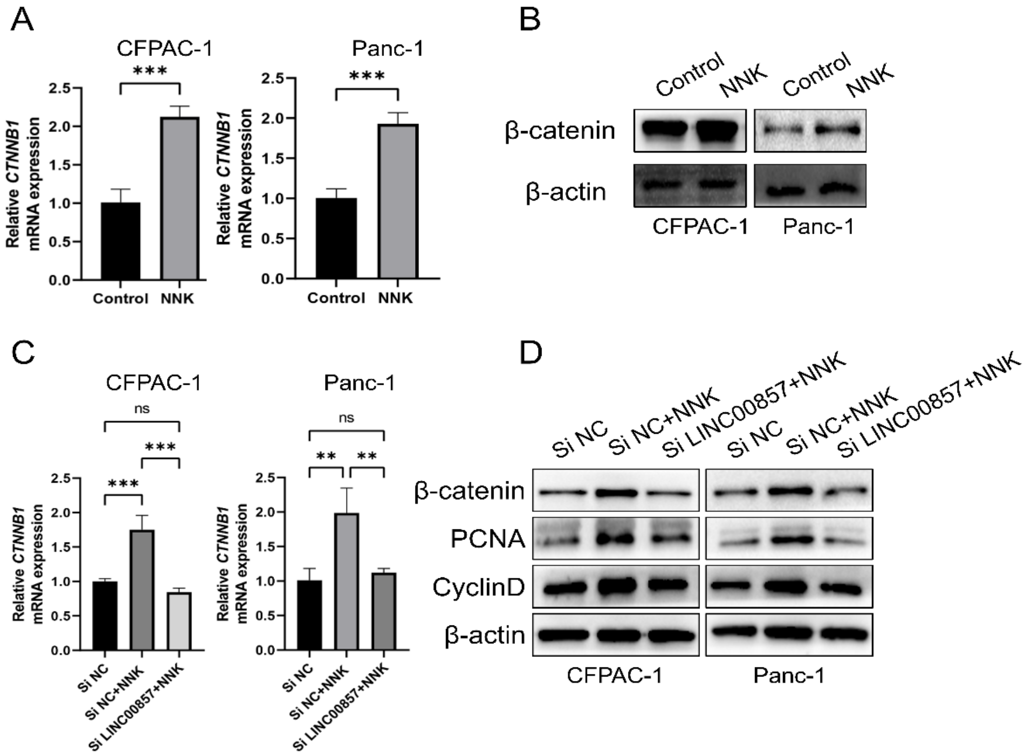

© 2025 Zhou C. et al.
